# Crystal and mol­ecular structures of a binuclear mixed ligand complex of silver(I) with thio­cyanate and 1*H*-1,2,4-triazole-5(4*H*)-thione

**DOI:** 10.1107/S2056989019016359

**Published:** 2020-01-01

**Authors:** Janjira Kreaunakpan, Kittipong Chainok, Nathan R. Halcovitch, Edward R. T. Tiekink, Teerapong Pirojsirikul, Saowanit Saithong

**Affiliations:** a Department of Chemistry, Faculty of Science, Prince of Songkla University, Hat Yai, Songkhla 90112, Thailand; b Materials and Textile Technology, Faculty of Science and Technology, Thammasat University, Khlong Luang, Pathum Thani, 12121, Thailand; c Department of Chemistry, Lancaster University, Lancaster LA1 4YB, United Kingdom; d Centre for Crystalline Materials, Faculty of Science and Technology, Sunway University, 47500 Bandar Sunway, Selangor Darul Ehsan, Malaysia; e Department of Chemistry and Center of Excellence for Innovation in Chemistry, Faculty of Science, Prince of Songkla University, Hat Yai, Songkhla 90112, Thailand

**Keywords:** crystal structure, Ag^I^ complex, hydrogen bonding, 1*H*-1,2,4-triazole-5(4-*H*–thione), thio­cyanate

## Abstract

The complete mol­ecule of the binuclear title complex, bis­[μ-1*H*-1,2,4-triazole-5(4*H*)-thione-κ^2^ *S*:*S*]bis­{(thio­cyanato-κ*S*)[1*H*-1,2,4-triazole-5(4*H*)-thione-κ*S*]silver(I)}, [Ag_2_(SCN)_2_(C_2_H_3_N_3_S)_4_], is generated by crystallographic inversion symmetry. The independent triazole-3-thione ligands employ the exocyclic-S atoms exclusively in coordination. One acts as a terminal S-ligand and the other in a bidentate (μ^2^) bridging mode to provide a link between two Ag^I^ centres. Each Ag^I^ atom is also coordinated by a terminal S-bound thio­cyanate ligand, resulting in a distorted AgS_4_ tetra­hedral coordination geometry. An intra­molecular N—H⋯S(thio­cyanate) hydrogen bond is noted. In the crystal, amine-N—H⋯S(thione), N—H⋯N(triazol­yl) and N—H⋯N(thio­cyanate) hydrogen bonds give rise to a three-dimensional architecture. The packing is consolidated by triazolyl-C—H⋯S(thio­cyanate), triazolyl-C—H⋯N(thiocyanate) and S⋯S [3.2463 (9) Å] inter­actions as well as face-to-face π–π stacking between the independent triazolyl rings [inter-centroid separation = 3.4444 (15) Å]. An analysis of the calculated Hirshfeld surfaces shows the three major contributors are due to N⋯H/H⋯N, S⋯H/H⋯S and C⋯H/H⋯C contacts, at 35.8, 19.4 and 12.7%, respectively; H⋯H contacts contribute only 7.6% to the overall surface.

## Abstract 2

The centrosymmetric binuclear complex features a tetra­hedral Ag^I^ centre within a S_4_ donor set. The three-dimensional mol­ecular packing is sustained by amine-*N*—*H*⋯*S*(thione), N—H⋯N(triazol­yl) and *N*—*H*⋯*N*(thio­cyanate) hydrogen bonds.

## Chemical context   

The title binuclear Ag^I^ complex, (I), containing 1*H*-1,2,4-triazole-5(4*H*-thione) and thio­cyanate ligands has been synthesized and its crystal and mol­ecular structures determined as part of our on-going studies in this area (Kodcharat *et al.*, 2013 ▸). Inter­est in the 1,2,4-triazole-based heterocyclic thione derives from the various medical applications and extensive biological activity exhibited by Schiff base mol­ecules derived from 1,2,4-triazoles. For example, these mol­ecules are known for their anti-fungal, anti-bacterial, anti-tumour, anti-convulsant, anti-inflammatory and analgesic properties (Al-Soud *et al.*, 2003 ▸; Walczak *et al.*, 2004 ▸; Almasirad *et al.*, 2004 ▸; Amir & Shikha, 2004 ▸; Turan-Zitouni *et al.*, 2005 ▸). In addition, the synthesis and biological activities of coordination complexes of these mol­ecules continue to attract significant attention as coordination often enhances the biological activity of the organic mol­ecules (Dharmaraj *et al.*, 2001 ▸; Singh *et al.*, 2006 ▸; Altundas *et al.*, 2010 ▸; Amer *et al.*, 2013 ▸; Bheeter *et al.*, 2016 ▸).
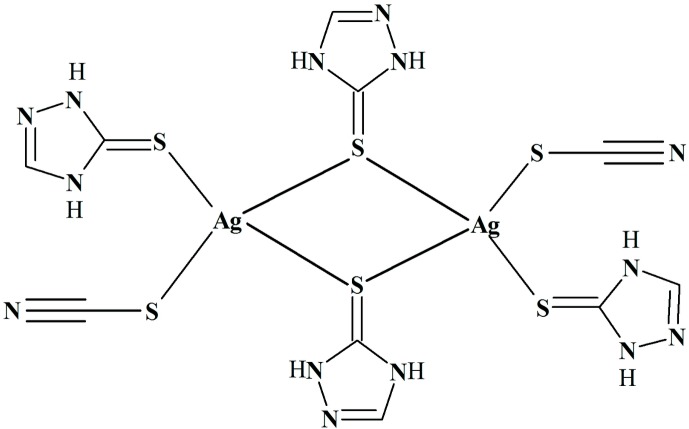


1*H*-1,2,4-Triazole-5(4*H-*thione), the heterocyclic ligand in (I) and hereafter referred to as HtrzSH, has attracted relatively little attention in the literature although recently the anti-cancer potential of derivatives of this were described (Büyükekşi *et al.*, 2018 ▸). The crystallographic study of (I) described herein is complemented by an analysis of the calculated HOMO and LUMO and an analysis of the calculated Hirshfeld surfaces and energy frameworks.

## Structural commentary   

**Figure 1:**
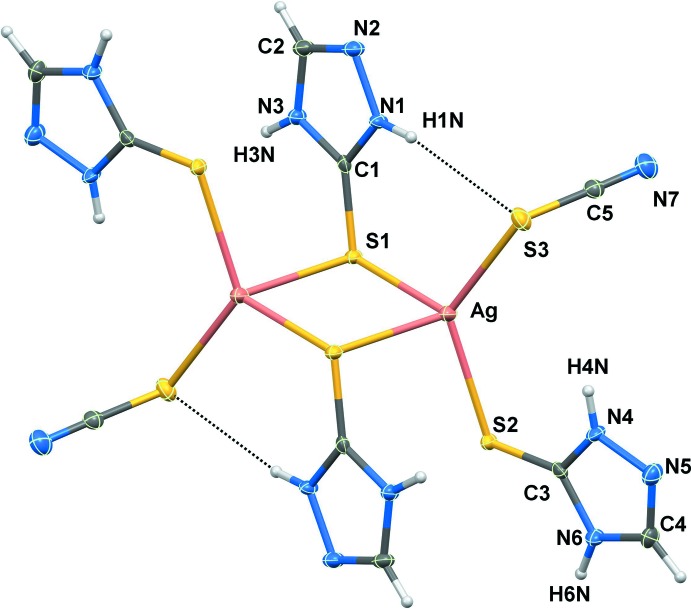
The mol­ecular structure of (I) showing displacement ellipsoids at the 70% probability level. The unlabelled atoms are generated by the symmetry operation 1 − *x*, 1 − *y*, 1 − *z*. The dashed lines represent intra­molecular amine-N—H⋯S(thio­cyanato) hydrogen bonds.

The binuclear complex, [Ag(HtrzSH)_2_(SCN)]_2_ (I), Fig. 1 ▸, crystallizes in the monoclinic space group *P*2_1_/*n* and is disposed about a crystallographic centre of inversion. The HtrzSH mol­ecules only employ their exocyclic thione-sulfur atoms in coordination, there being no Ag⋯N contacts of note. Each Ag^I^ atom is coordinated by a terminally bound HtrzSH mol­ecule and by two thione-sulfur atoms derived from two μ_2_-bridging HtrzSH mol­ecules. The coordination of each Ag^I^ atom is completed by a terminal, S-bound thio­cyanate anion. The geometry around the silver centre defined by the S_4_ donor set is distorted tetra­hedral with the S—Ag—S bond angles spanning about 25°, *i.e*. from a narrow 91.60 (2)° for S1—Ag—S1^i^, being subtended by the bridging S1 atoms, to a wide 127.43 (2)° for S2—Ag—S3; symmetry operation (i): 1 − *x*, 1 − *y*, 1 − *z*. The Ag_2_S_2_ core has the shape of a distorted rhombus as the Ag—S1 bond length of 2.5596 (7) Å is significantly shorter than the Ag—S1^i^ bond of 2.8188 (7) Å. The Ag—S bond lengths fall in two distinct classes, with the Ag—S1_b_ and Ag—S_t_ (b = bridging, t = thio­cyanate) bond lengths being similar and shorter than Ag—S1^i^_b_ (Table 1 ▸). Despite the different modes of coordination of the thione-S atoms, the C1—S1 and C3—S2 bond lengths are indistinguishable at 1.698 (3) Å. Each of the C1—S1 and C3—S2 bond lengths in (I) are marginally longer than 1.6836 (19) Å found in the structure of the free mol­ecule (Büyükekşi *et al.*, 2018 ▸). This small difference is reflected in the observation that no significant differences are evident in bond lengths within the five-membered rings in (I) and those in the uncomplexed mol­ecule (Büyükekşi *et al.*, 2018 ▸).

The five-membered rings lie prime to either side of the Ag_2_S_2_ core, with the dihedral angles between the core and the N1- and N4-rings being 88.99 (11) and 85.16 (11)°, respectively. The independent rings are close to being co-planar, exhibiting a dihedral angle of 8.38 (16)°. Finally, the N1-amine is orientated to be in close proximity to the S3-thio­cyanato atom, enabling the formation of an intra­molecular amine-N—H⋯S(thio­cyanato) hydrogen bond (Table 2 ▸). While the N4-amine is similarly oriented, the H⋯S separation of 3.31 Å is not indicative of a significant inter­action.

## Supra­molecular features   

**Figure 2:**
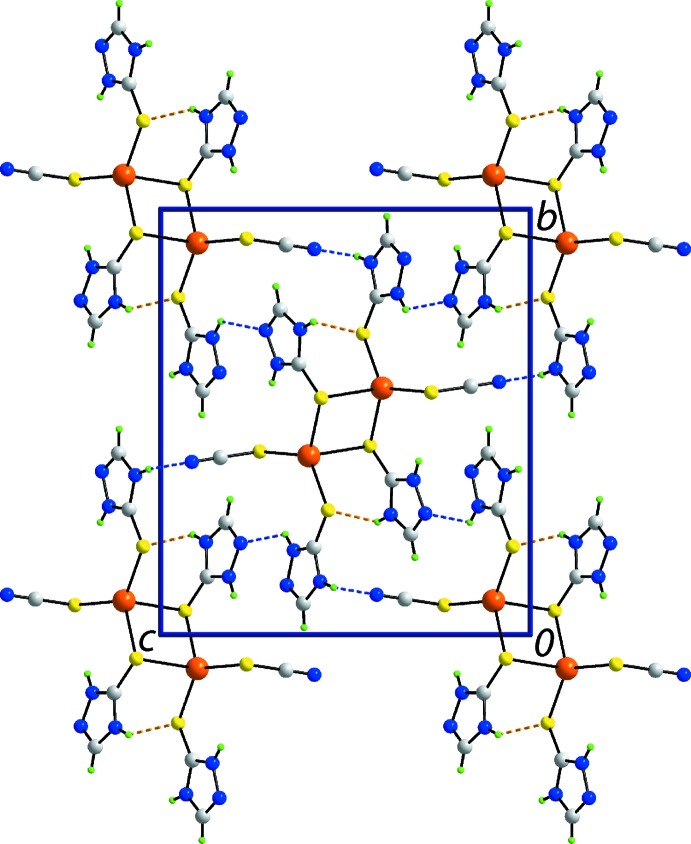
A view of the unit-cell contents of (I) in projection down the *a* axis, with N—H⋯S and N—H⋯N hydrogen bonds shown as orange and blue dashed lines, respectively.

**Figure 3:**
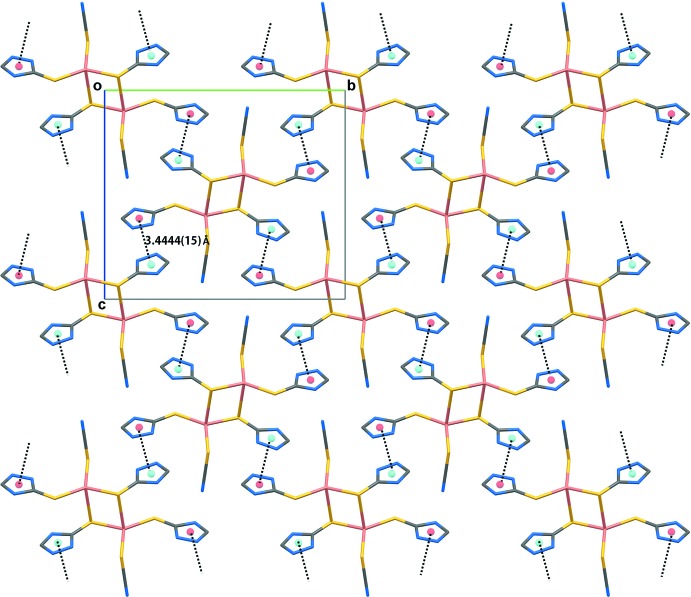
The face-to-face π–π stacking of (I).

The crystal of (I) consists of a three-dimensional network of hydrogen bonds and other non-covalent contacts as summarized in Table 2 ▸. The second amine-N—H atom of the S1-thione mol­ecule [the first is engaged in an intra­molecular N—H⋯S(thio­cyanate) hydrogen bond and a second, weaker N—H⋯N5(triazol­yl) inter­action] forms a hydrogen bond to the thione-S2 atom. By contrast, the amine-N—H atoms of the S2-thione mol­ecule form N—H⋯N(triazol­yl) and N—H⋯N(thio­cyanate) hydrogen bonds. The hydrogen bonds combine to sustain a three-dimensional architecture as shown in Fig. 2 ▸. Further stability to the mol­ecular packing is provided by triazolyl-C—H⋯S(thio­cyanate) and triazolyl-C—H⋯N(thio­cyanate) inter­actions along with face-to-face π–π stacking (Fig. 3 ▸). The latter occur between the independent triazolyl rings [inter-centroid separation: (N1–N3,C1,C2)⋯(N4–N6,C3,C4)^ii^ = 3.4444 (15) Å and angle of inclination = 6.81 (16)° for (ii) 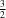 − *x*, 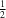 + *y*, 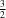 − *z*].

## Analysis of the Hirshfeld surfaces   

The Hirshfeld surface analysis (McKinnon *et al.*, 2004 ▸; Tan *et al.*, 2019 ▸) of (I) was performed using *Crystal Explorer 17* (Turner *et al.*, 2017 ▸) to give further insight into the important inter­molecular contacts normalized by van der Waals radii through a red–white–blue surface colour scheme where these colours denote the close contacts shorter than, equal to and longer than the sun of the respective van der Waals radii.

**Figure 4:**
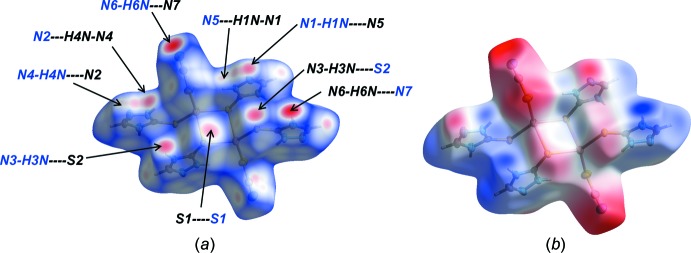
A view of the Hirshfeld surface for (I) mapped over (*a*) *d*_norm_ and (*b*) the electrostatic potential; the red and blue regions represent negative and positive electrostatic potentials, respectively.

**Figure 5:**
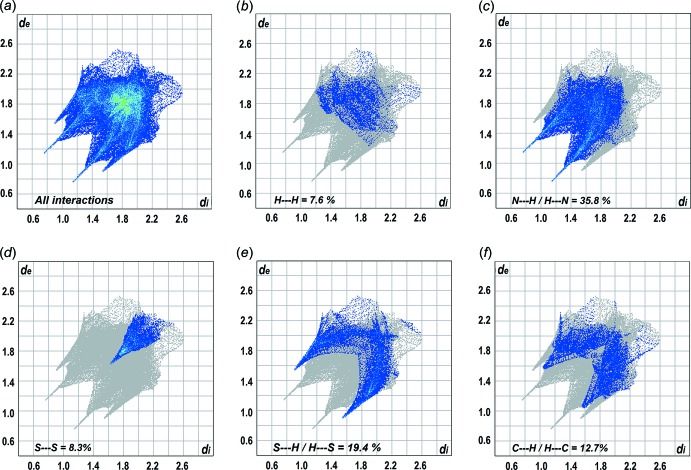
(*a*) A comparison of the full two-dimensional fingerprint plot for (I) and those delineated into (*b*) H⋯H, (*c*) N⋯H/H⋯N, (*d*) S⋯S, (*e*) S⋯H/H⋯S and (*f*) C⋯H/H⋯C contacts.

As seen in Fig. 4 ▸(*a*) of the Hirshfeld surface plotted over *d*_norm_ for (I), the red regions of the surface represent close contacts corresponding to the N—H⋯S and N—H⋯N hydrogen-bonding inter­actions mentioned above. An additional feature, *i.e*. S⋯S contacts, are noted. The closest of these, *i.e*. S1⋯S1^iii^ = 3.2463 (9) Å [symmetry operation: (iii) 2 − *x*, 1 − *y*, 1 − *z*], link the binuclear mol­ecules into chains along the *a*-axis direction. On the Hirshfeld surface mapped over electrostatic potential (DFT 3-21G) shown in Fig. 4 ▸(*b*), the faint-red and light-blue regions correspond to negative and positive electrostatic potential, respectively,

The full and delineated (H⋯H, N⋯H/H⋯N, S⋯S, S⋯H/H⋯S and C⋯H/H⋯C) two-dimensional fingerprint plots are shown in Fig. 5 ▸(*a*)–(*f*), respectively. The N⋯H/H⋯N contacts, at 35.8%, are the major contributor to the Hirshfeld surface. The S⋯H/H⋯S contacts (19.4%) also make a significant contribution. Other significant contributions come from the C⋯H/H⋯C (12.7%) and S⋯S (8.3%) contacts with H⋯H contacts, occurring at distances beyond the sum of the van der Waals radii, contributing only 7.6%. The next most significant contribution is made by N⋯C/C⋯N contacts (6.7%) arising in the main from the π–π stacking inter­actions between triazolyl rings.

The energy frameworks were simulated (Turner *et al.*, 2017 ▸) in order to analyse the specific inter­molecular inter­actions identified above for each mol­ecule-to-mol­ecule contact. This was achieved by summing up four different energy components (Turner *et al.*, 2017 ▸) for each pair of mol­ecules, *i.e*. electrostatic (*E*_ele_), polarization (*E*_pol_), dispersion (*E*_dis_) and exchange–repulsion (*E*_rep_); these were obtained using the wave function calculated at the HF/3-21G level of theory. The results are summarized in Table 3 ▸. The greatest energy of attraction between mol­ecules amounts to 138.4 kJ mol^−1^, having a major electrostatic contribution (−142.4 kJ mol^−1^), and is associated with the following inter­atomic contacts: C2—H2⋯N7, C4—H4⋯N7 and π–π stacking of between triazole rings. The next most significant contribution, with a total energy of −125.0 kJ mol^−1^, arises from conventional hydrogen bonds, *i.e*. N1—H1*N*⋯N5, N4—H4*N*⋯N2 and N6—H6*N*⋯N7 as well as C2—H2⋯S3 inter­actions. The next attractive inter­action, with *E*_tot_ = −48.9 and *E*_dis_ = −120.3 kJ mol^−1^, respectively, reflects the N3—H3*N*⋯S2 hydrogen bonding and S1⋯S1 secondary bonding contact.

**Figure 6:**
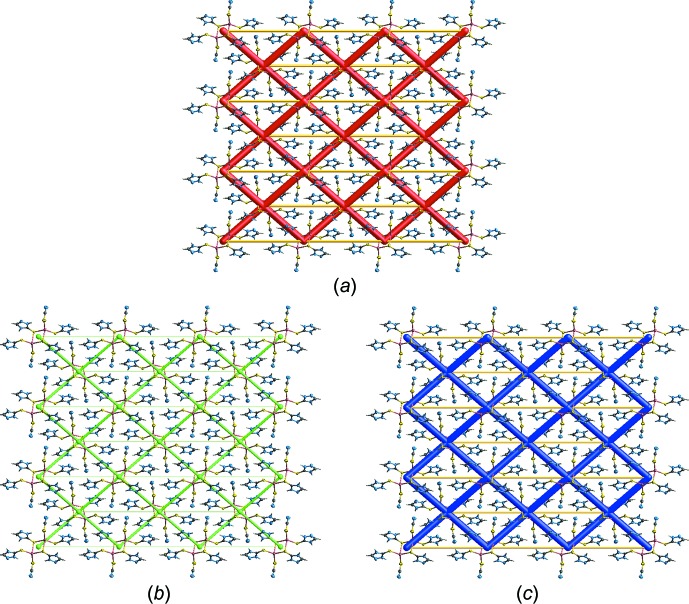
The colour inter­action mapping and energy frameworks for (I) showing the (*a*) electrostatic potential force, (*b*) dispersion force and (*c*) total energy diagrams. All cylindrical radii were adjusted to the same scale factor of 100 with a cut-off value of −50.0 kJ mol^−1^ within a 3 × 3 × 3 unit cell and their sizes are proportional to the relative strength of the corresponding energies.

The magnitudes of inter­molecular energies, *i.e*. the *E*_ele_, *E*_dis_ and *E*_tot_ components, are represented graphically in Fig. 6 ▸(*a*)–(*c*), respectively, by energy framework diagrams whereby the cylinders join the centroids of mol­ecular pairs using a red, green and blue colour scheme; the radius of the cylinder is proportional to the magnitude of inter­action energy.

## Mol­ecular orbital calculations   

The HOMO and LUMO energies for the atom positions in the crystal structure of (I) were calculated using a pseudo-potential plane-wave DFT method (Parr & Yang, 1994 ▸) implemented in the *NWChem* package (Valiev *et al.*, 2010 ▸). The plane wave basis set and PBE exchange-correlation functional were chosen for the calculations on the experimental structure, *i.e*. without geometry optimization. The HOMO and LUMO of (I) are illustrated in Fig. S1 in the supporting information and their energies were calculated to be 3.011 and 6.173 eV, respectively. The HOMO is delocalized across the thio­cyanato groups and the bridging region between the two dimers. The LUMO includes the delocalization around the triazole rings.

## Database survey   

A survey of the Cambridge Structural Database (Groom *et al.*, 2016 ▸) for coordination complexes of HtrzSH yielded seven structures. Monodentate coordination *via* the thione-S atom, as in (I), has been identified in six structures. The first three of these are neutral and mononuclear, namely [(Ph_3_P)_2_Cu(HtrzSH)Cl]·CH_3_CN, (NEPPOP; Wani *et al.*, 2013 ▸), [Ag(HtrzSH)(NO_3_)]·CH_3_OH (GISHUN; Wattanakanjana *et al.*, 2014 ▸) and [Cd(HtrzSH)(H_2_Edta)]·H_2_O (LOFKAT; Zhang *et al.*, 2008 ▸); H_4_Edta is ethyl­enedi­amine tetra­carb­oxy­lic acid. The mononuclear Fe^III^ complex, Fe(NO)_2_(HtrzS)(HtrzSH)·0.5H_2_O (EYABOV01; Aldoshin *et al.*, 2008 ▸) contains both neutral and mono-anionic forms of HtrzSH. The fifth structure featuring monodentate coordination of HtrzSH is a two-dimensional coordination polymer, *i.e*. {[Cd_2_(O_2_CCO_2_)_2_(HtrzSH)_2_]·2H_2_O}_*n*_ (ZIVBOX; Liang *et al.*, 2014 ▸); there are two distinct Cd^II^ atom coordination environments. Two distinct coordination modes for HtrzSH are noted in the structure of [Cd(HtrzSH)_2_Cl_2_]_*n*_ (LOFJEW; Zhang *et al.*, 2008 ▸), *i.e.* monodentate, as for the above, as well as bidentate, μ_2_-bridging as one of the triazolyl-N atoms also coordinates Cd^II^ in this polymeric structure. In the final structure with HtrzSH, tridentate coordination for HtrzSH *via* the thione-S atom only has been observed in [Ag(HtrzSH)Cl]_*n*_ (XINDUV; Kang *et al.*, 2013 ▸), which is a two-dimensional coordination polymer.

As indicated above for Fe(NO)_2_(HtrzS)(HtrzSH)·0.5H_2_O (EYABOV01; Aldoshin *et al.*, 2008 ▸), mono-anionic forms of HtrzSH are known. Here, HtrzS functions as a monodentate thiol­ate-S ligand. A monodentate thiol­ate-S mode of coord­ination is also seen in (3-ClC_6_H_4_CH_2_)_3_Sn(HtrzS) (SUXSAG; Keng *et al.*, 2010 ▸). The three remaining structures feature a tridentate coordination mode leading to coordination polymers. In [Cu(HtrzS)]_*n*_ (TEHYIQ; Zhang *et al.*, 2012 ▸), this is achieved by bidentate, *μ_2_*-bridging by the thiol­ate-S atom and the participation of one of the triazolyl-N atoms in coordin­ation. In [Pb(HtrzS)(NO_3_)OH_2_]_*n*_ (MOKKAA; Imran *et al.*, 2015 ▸), the thiol­ate-S and two triazolyl-N atoms are involved in coordination. A similar coordination mode is found for one of the independent anions in [Cd_2_(HtrzS)_2_(SO_4_)]_n_ (LOFJUM; Zhang *et al.*, 2008 ▸). The second anion is tetra­dentate as the thiol­ate-S atom is bidentate, *μ_2_*-bridging. From the foregoing, it is evident that HtrzSH/HtrzS ligands adopt a wide range of coordination modes in the relatively few structures in which they have been characterized, suggesting further work in this area is warranted.

## Synthesis and crystallization   

Silver nitrate (0.21 g, 1.24 mmol) and potassium thio­cyanate (0.12 g, 1.23 mmol) were dissolved in acetro­nitrile (25 ml) and a white precipitate formed. This mixture was heated at 323–325 K for 30 min. Then, a clear solution of 1*H*-1,2,4-triazole-3-thiol (0.25 g, 2.47 mmol) in distilled water (5 ml) was added followed by heating for 4.3 h during which time the precipitate slowly dissolved. The clear solution was filtered and kept to evaporate at ambient temperature. After a few days, colourless trapezoidal prisms of (I) formed, which were filtered off and dried *in vacuo*. M.p.: 413–417 K. IR (solid KBr pellet, cm^−1^): 2108 (*s*) (C≡N), 1479 (*s*) (C=N), 1248 (*w*) (C–N), 1054 (*m*) (C—S) + (C—N).

## Refinement   

Crystal data, data collection and structure refinement details are summarized in Table 4 ▸. The H atoms were found in difference Fourier maps and their positions refined resulting in distances of N—H = 0.80 (4)–0.877 (10) Å and C—H = 0.89 (4)–0.91 (4) Å, and with *U*_iso_(H) = 1.2*U*_eq_(N, C). The maximum and minimum residual electron density peaks of 0.96 and 1.04 e Å^−3^, respectively, were located 0.83 and 0.77 Å from the N3 and Ag atoms, respectively.

## Supplementary Material

Crystal structure: contains datablock(s) I. DOI: 10.1107/S2056989019016359/hb7868sup1.cif

Structure factors: contains datablock(s) I. DOI: 10.1107/S2056989019016359/hb7868Isup2.hkl

Fig. S1 Image of the calculated HOMO and LUMO's for (I). DOI: 10.1107/S2056989019016359/hb7868sup3.tif

CCDC reference: 1969873

Additional supporting information:  crystallographic information; 3D view; checkCIF report

## Figures and Tables

**Table 1:** Selected geometric parameters (Å, °)

Ag—S1	2.5596 (7)	C1—S1	1.698 (3)
Ag—S2	2.5103 (6)	C3—S2	1.698 (3)
Ag—S3	2.5374 (7)	C5—S3	1.660 (3)
Ag—S1^i^	2.8188 (7)		
			
S1—Ag—S2	112.49 (2)	S2—Ag—S3	127.43 (2)
S1—Ag—S3	114.64 (2)	S2—Ag—S1^i^	99.56 (2)
S1—Ag—S1^i^	91.60 (2)	S3—Ag—S1^i^	101.08 (2)

Symmetry code: (i) 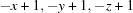.

**Table 2:** Hydrogen-bond geometry (Å, °)

*D*—H⋯*A*	*D*—H	H⋯*A*	*D*⋯*A*	*D*—H⋯*A*
N1—H1*N*⋯S3	0.88 (1)	2.72 (2)	3.555 (2)	161 (3)
N1—H1*N*⋯N5^ii^	0.88 (3)	2.57 (4)	3.023 (3)	113
N3—H3*N*⋯S2^iii^	0.88 (1)	2.56 (2)	3.345 (2)	150 (3)
N4—H4*N*⋯N2^iv^	0.80 (4)	2.38 (4)	2.900 (3)	123 (3)
N6—H6*N*⋯N7^v^	0.88 (1)	2.03 (1)	2.877 (3)	163 (3)
C2—H2⋯S3^ii^	0.89 (4)	2.87 (4)	3.504 (3)	129 (3)
C2—H2⋯N7^vi^	0.89 (4)	2.66 (3)	3.184 (4)	118 (3)
C4—H4⋯N7^vii^	0.91 (4)	2.58 (4)	3.306 (4)	137 (3)

Symmetry codes: (ii) 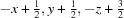; (iii) 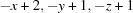; (iv) 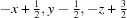; (v) 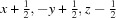; (vi) 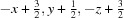; (vii) 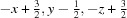.

**Table 3:** Summary of inter­action energies (kJ mol^−1^) calculated for (I)

*R* (Å)	*E*_ele_	*E*_pol_	*E*_dis_	*E*_rep_	*E*_tot_	Symmetry operation
11.06	−142.4	−31.6	−39.9	77.9	−138.4	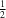 − *x*, 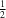 + *y*, 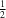 − *z*
10.68	−89.0	−31.2	−54.8	43.5	−125.0	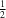 − *x*, 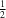 + *y*, 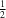 − *z*
4.87	−50.3	−50.1	−120.3	176.8	−48.9	*x*, *y*, *z*
16.68	22.9	−3.0	−1.8	0.0	19.8	*x*, *y*, *z*
15.95	42.9	−7.4	−7.5	0.8	32.8	*x*, *y*, *z*

**Table 4:** Experimental details

Crystal data	
Chemical formula	[Ag_2_(SCN)_2_(C_2_H_3_N_3_S)_4_]
*M*_r_	736.44
Crystal system, space group	Monoclinic, *P*2_1_/*n*
Temperature (K)	100
*a*, *b*, *c* (Å)	4.8718 (1), 15.9511 (1), 13.9575 (1)
β (°)	96.945 (1)
*V* (Å^3^)	1076.69 (2)
*Z*	2
Radiation type	Cu *K*α
μ (mm^−1^)	20.35
Crystal size (mm)	0.41 × 0.14 × 0.11

Data collection	
Diffractometer	Rigaku Oxford Diffraction SuperNova, Dual, Cu at zero, AtlasS2
Absorption correction	Gaussian (*CrysAlis PRO*; Rigaku OD, 2015 ▸)
*T*_min_, *T*_max_	0.097, 0.453
No. of measured, independent and observed [*I* > 2σ(*I*)] reflections	18122, 2266, 2242
*R*_int_	0.039
(sin θ/λ)_max_ (Å^−1^)	0.633

Refinement	
*R*[*F* ^2^ > 2σ(*F* ^2^)], *wR*(*F* ^2^), *S*	0.025, 0.066, 1.17
No. of reflections	2266
No. of parameters	163
No. of restraints	10
H-atom treatment	Only H-atom coordinates refined
Δρ_max_, Δρ_min_ (e Å^−3^)	0.96, −1.04

Computer programs: *CrysAlis PRO* (Rigaku OD, 2015 ▸), *SHELXT* (Sheldrick, 2015 ▸), *SHELXL2014/7* (Sheldrick, 2015 ▸), *Mercury* (Macrae *et al.*, 2008 ▸), *DIAMOND* (Brandenburg, 2006 ▸), *WinGX* (Farrugia, 2012 ▸) and *publCIF* (Westrip, 2010 ▸).

## supplementary crystallographic information

### Crystal data

**Table:**

[Ag_2_(SCN)_2_(C_2_H_3_N_3_S)_4_]	*F*(000) = 720
*M**_r_* = 736.44	*D*_x_ = 2.272 Mg m^−^^3^
Monoclinic, *P*2_1_/*n*	Cu *K*α radiation, λ = 1.54184 Å
*a* = 4.8718 (1) Å	Cell parameters from 13766 reflections
*b* = 15.9511 (1) Å	θ = 3.2–77.1°
*c* = 13.9575 (1) Å	µ = 20.35 mm^−^^1^
β = 96.945 (1)°	*T* = 100 K
*V* = 1076.69 (2) Å^3^	Prism, colourless
*Z* = 2	0.41 × 0.14 × 0.11 mm

### Data collection

**Table:**

Rigaku Oxford Diffraction SuperNova, Dual, Cu at zero, AtlasS2 diffractometer	2266 independent reflections
Mirror monochromator	2242 reflections with *I* > 2σ(*I*)
Detector resolution: 5.2303 pixels mm^-1^	*R*_int_ = 0.039
ω scans	θ_max_ = 77.4°, θ_min_ = 4.2°
Absorption correction: gaussian (CrysAlisPro; Rigaku OD, 2015)	*h* = −6→5
*T*_min_ = 0.097, *T*_max_ = 0.453	*k* = −19→20
18122 measured reflections	*l* = −17→17

### Refinement

**Table:**

Refinement on *F*^2^	Primary atom site location: structure-invariant direct methods
Least-squares matrix: full	Secondary atom site location: difference Fourier map
*R*[*F*^2^ > 2σ(*F*^2^)] = 0.025	Hydrogen site location: difference Fourier map
*wR*(*F*^2^) = 0.066	Only H-atom coordinates refined
*S* = 1.17	*w* = 1/[σ^2^(*F*_o_^2^) + (0.0343*P*)^2^ + 1.905*P*] where *P* = (*F*_o_^2^ + 2*F*_c_^2^)/3
2266 reflections	(Δ/σ)_max_ = 0.001
163 parameters	Δρ_max_ = 0.96 e Å^−^^3^
10 restraints	Δρ_min_ = −1.04 e Å^−^^3^

### Special details

**Table:**

Geometry. All esds (except the esd in the dihedral angle between two l.s. planes) are estimated using the full covariance matrix. The cell esds are taken into account individually in the estimation of esds in distances, angles and torsion angles; correlations between esds in cell parameters are only used when they are defined by crystal symmetry. An approximate (isotropic) treatment of cell esds is used for estimating esds involving l.s. planes.
Refinement. C5 treated with ISOR

### Fractional atomic coordinates and isotropic or equivalent isotropic displacement parameters (Å^2^)

**Table:**

	*x*	*y*	*z*	*U*_iso_*/*U*_eq_	
Ag	0.56775 (4)	0.42032 (2)	0.59899 (2)	0.01119 (9)	
S1	0.81885 (13)	0.55635 (4)	0.56609 (4)	0.00788 (14)	
S2	0.81257 (13)	0.29260 (4)	0.54731 (4)	0.00780 (14)	
S3	0.25779 (15)	0.43047 (4)	0.73213 (5)	0.01162 (15)	
N1	0.4853 (5)	0.63635 (14)	0.68422 (16)	0.0077 (4)	
H1N	0.390 (6)	0.5923 (15)	0.698 (3)	0.009*	
N2	0.4273 (5)	0.71516 (14)	0.71587 (16)	0.0097 (4)	
N3	0.7598 (5)	0.71790 (14)	0.62260 (16)	0.0082 (4)	
H3N	0.885 (5)	0.735 (2)	0.587 (2)	0.010*	
N4	0.4504 (5)	0.19814 (14)	0.63991 (16)	0.0087 (4)	
H4N	0.369 (8)	0.236 (2)	0.663 (3)	0.010*	
N5	0.3865 (5)	0.11639 (15)	0.65884 (17)	0.0117 (5)	
N6	0.7280 (5)	0.12487 (14)	0.56881 (16)	0.0089 (4)	
H6N	0.854 (5)	0.111 (2)	0.532 (2)	0.011*	
N7	0.5604 (6)	0.40606 (16)	0.91561 (18)	0.0150 (5)	
C1	0.6846 (5)	0.63623 (16)	0.62603 (18)	0.0070 (5)	
C2	0.5982 (6)	0.76322 (17)	0.67677 (19)	0.0093 (5)	
H2	0.615 (7)	0.818 (2)	0.688 (2)	0.011*	
C3	0.6577 (6)	0.20539 (16)	0.58544 (18)	0.0076 (5)	
C4	0.5594 (6)	0.07362 (17)	0.6139 (2)	0.0110 (6)	
H4	0.571 (7)	0.017 (3)	0.612 (2)	0.013*	
C5	0.4422 (6)	0.41636 (15)	0.8389 (2)	0.0089 (5)	

### Atomic displacement parameters (Å^2^)

**Table:**

	*U*^11^	*U*^22^	*U*^33^	*U*^12^	*U*^13^	*U*^23^
Ag	0.01970 (15)	0.00638 (12)	0.00859 (12)	0.00126 (7)	0.00616 (8)	−0.00024 (6)
S1	0.0112 (3)	0.0055 (3)	0.0077 (3)	0.0013 (2)	0.0041 (2)	−0.0004 (2)
S2	0.0108 (3)	0.0057 (3)	0.0078 (3)	0.0001 (2)	0.0048 (2)	0.0008 (2)
S3	0.0137 (4)	0.0139 (3)	0.0078 (3)	−0.0011 (2)	0.0037 (2)	0.0010 (2)
N1	0.0106 (11)	0.0055 (10)	0.0080 (10)	−0.0009 (8)	0.0056 (8)	−0.0010 (8)
N2	0.0125 (12)	0.0072 (10)	0.0101 (11)	−0.0003 (9)	0.0043 (9)	−0.0023 (8)
N3	0.0112 (12)	0.0057 (10)	0.0085 (10)	−0.0012 (8)	0.0046 (8)	−0.0004 (8)
N4	0.0116 (12)	0.0047 (10)	0.0112 (11)	0.0016 (9)	0.0063 (9)	0.0005 (8)
N5	0.0142 (12)	0.0078 (11)	0.0139 (11)	−0.0003 (9)	0.0053 (9)	0.0018 (8)
N6	0.0113 (12)	0.0070 (10)	0.0094 (10)	0.0019 (9)	0.0051 (8)	0.0002 (8)
N7	0.0191 (14)	0.0151 (11)	0.0118 (12)	−0.0019 (10)	0.0064 (10)	0.0013 (9)
C1	0.0087 (13)	0.0075 (12)	0.0045 (11)	0.0001 (9)	0.0001 (9)	0.0014 (9)
C2	0.0118 (14)	0.0069 (12)	0.0097 (12)	−0.0002 (10)	0.0029 (10)	−0.0026 (9)
C3	0.0096 (13)	0.0090 (12)	0.0042 (11)	0.0016 (10)	0.0007 (9)	0.0000 (9)
C4	0.0154 (16)	0.0070 (13)	0.0111 (13)	−0.0016 (10)	0.0034 (11)	0.0017 (9)
C5	0.0093 (9)	0.0086 (8)	0.0096 (9)	−0.0006 (7)	0.0041 (7)	−0.0005 (7)

### Geometric parameters (Å, º)

**Table:**

Ag—S1	2.5596 (7)	N3—C2	1.363 (3)
Ag—S2	2.5103 (6)	N3—H3N	0.877 (10)
Ag—S3	2.5374 (7)	N4—C3	1.341 (3)
Ag—S1^i^	2.8188 (7)	N4—N5	1.374 (3)
C1—S1	1.698 (3)	N4—H4N	0.80 (4)
S1—Ag^i^	2.8188 (7)	N5—C4	1.302 (4)
C3—S2	1.698 (3)	N6—C3	1.357 (3)
C5—S3	1.660 (3)	N6—C4	1.365 (4)
N1—C1	1.339 (3)	N6—H6N	0.878 (7)
N1—N2	1.373 (3)	N7—C5	1.164 (4)
N1—H1N	0.878 (10)	C2—H2	0.89 (4)
N2—C2	1.299 (4)	C4—H4	0.91 (4)
N3—C1	1.356 (3)		
			
S1—Ag—S2	112.49 (2)	C3—N4—H4N	127 (3)
S1—Ag—S3	114.64 (2)	N5—N4—H4N	120 (3)
S1—Ag—S1^i^	91.60 (2)	C4—N5—N4	103.3 (2)
S2—Ag—S3	127.43 (2)	C3—N6—C4	108.0 (2)
S2—Ag—S1^i^	99.56 (2)	C3—N6—H6N	123 (2)
S3—Ag—S1^i^	101.08 (2)	C4—N6—H6N	128 (2)
C1—S1—Ag	109.08 (9)	N1—C1—N3	103.8 (2)
C1—S1—Ag^i^	92.55 (9)	N1—C1—S1	130.6 (2)
Ag—S1—Ag^i^	88.40 (2)	N3—C1—S1	125.6 (2)
C3—S2—Ag	109.28 (9)	N2—C2—N3	111.3 (2)
C5—S3—Ag	110.08 (10)	N2—C2—H2	124 (2)
C1—N1—N2	112.9 (2)	N3—C2—H2	124 (2)
C1—N1—H1N	125 (2)	N4—C3—N6	103.8 (2)
N2—N1—H1N	122 (2)	N4—C3—S2	129.9 (2)
C2—N2—N1	103.8 (2)	N6—C3—S2	126.2 (2)
C1—N3—C2	108.3 (2)	N5—C4—N6	111.6 (2)
C1—N3—H3N	122 (2)	N5—C4—H4	126 (2)
C2—N3—H3N	130 (2)	N6—C4—H4	122 (2)
C3—N4—N5	113.3 (2)	N7—C5—S3	176.9 (3)
			
C1—N1—N2—C2	−0.8 (3)	N1—N2—C2—N3	−0.2 (3)
C3—N4—N5—C4	−0.4 (3)	C1—N3—C2—N2	1.0 (3)
N2—N1—C1—N3	1.4 (3)	N5—N4—C3—N6	0.2 (3)
N2—N1—C1—S1	−177.4 (2)	N5—N4—C3—S2	−177.8 (2)
C2—N3—C1—N1	−1.4 (3)	C4—N6—C3—N4	0.1 (3)
C2—N3—C1—S1	177.5 (2)	C4—N6—C3—S2	178.2 (2)
Ag—S1—C1—N1	1.2 (3)	Ag—S2—C3—N4	−4.3 (3)
Ag^i^—S1—C1—N1	90.4 (3)	Ag—S2—C3—N6	178.2 (2)
Ag—S1—C1—N3	−177.4 (2)	N4—N5—C4—N6	0.4 (3)
Ag^i^—S1—C1—N3	−88.2 (2)	C3—N6—C4—N5	−0.4 (3)

Symmetry code: (i) −*x*+1, −*y*+1, −*z*+1.

### Hydrogen-bond geometry (Å, º)

**Table:**

*D*—H···*A*	*D*—H	H···*A*	*D*···*A*	*D*—H···*A*
N1—H1*N*···S3	0.88 (1)	2.72 (2)	3.555 (2)	161 (3)
N1—H1*N*···N5^ii^	0.88 (3)	2.57 (4)	3.023 (3)	113
N3—H3*N*···S2^iii^	0.88 (1)	2.56 (2)	3.345 (2)	150 (3)
N4—H4*N*···N2^iv^	0.80 (4)	2.38 (4)	2.900 (3)	123 (3)
N6—H6*N*···N7^v^	0.88 (1)	2.03 (1)	2.877 (3)	163 (3)
C2—H2···S3^ii^	0.89 (4)	2.87 (4)	3.504 (3)	129 (3)
C2—H2···N7^vi^	0.89 (4)	2.66 (3)	3.184 (4)	118 (3)
C4—H4···N7^vii^	0.91 (4)	2.58 (4)	3.306 (4)	137 (3)

Symmetry codes: (ii) −*x*+1/2, *y*+1/2, −*z*+3/2; (iii) −*x*+2, −*y*+1, −*z*+1; (iv) −*x*+1/2, *y*−1/2, −*z*+3/2; (v) *x*+1/2, −*y*+1/2, *z*−1/2; (vi) −*x*+3/2, *y*+1/2, −*z*+3/2; (vii) −*x*+3/2, *y*−1/2, −*z*+3/2.
