# Supplementary figures and images for: Crystal and mol­ecular structures of a binuclear mixed ligand complex of silver(I) with thio­cyanate and 1H-1,2,4-triazole-5(4H)-thione
Source: Acta Crystallogr E Crystallogr Commun. 2020 Jan 1;76(Pt 1):42–7. doi: 10.1107/S2056989019016359 (PMC6944082; doi:10.1107/S2056989019016359)

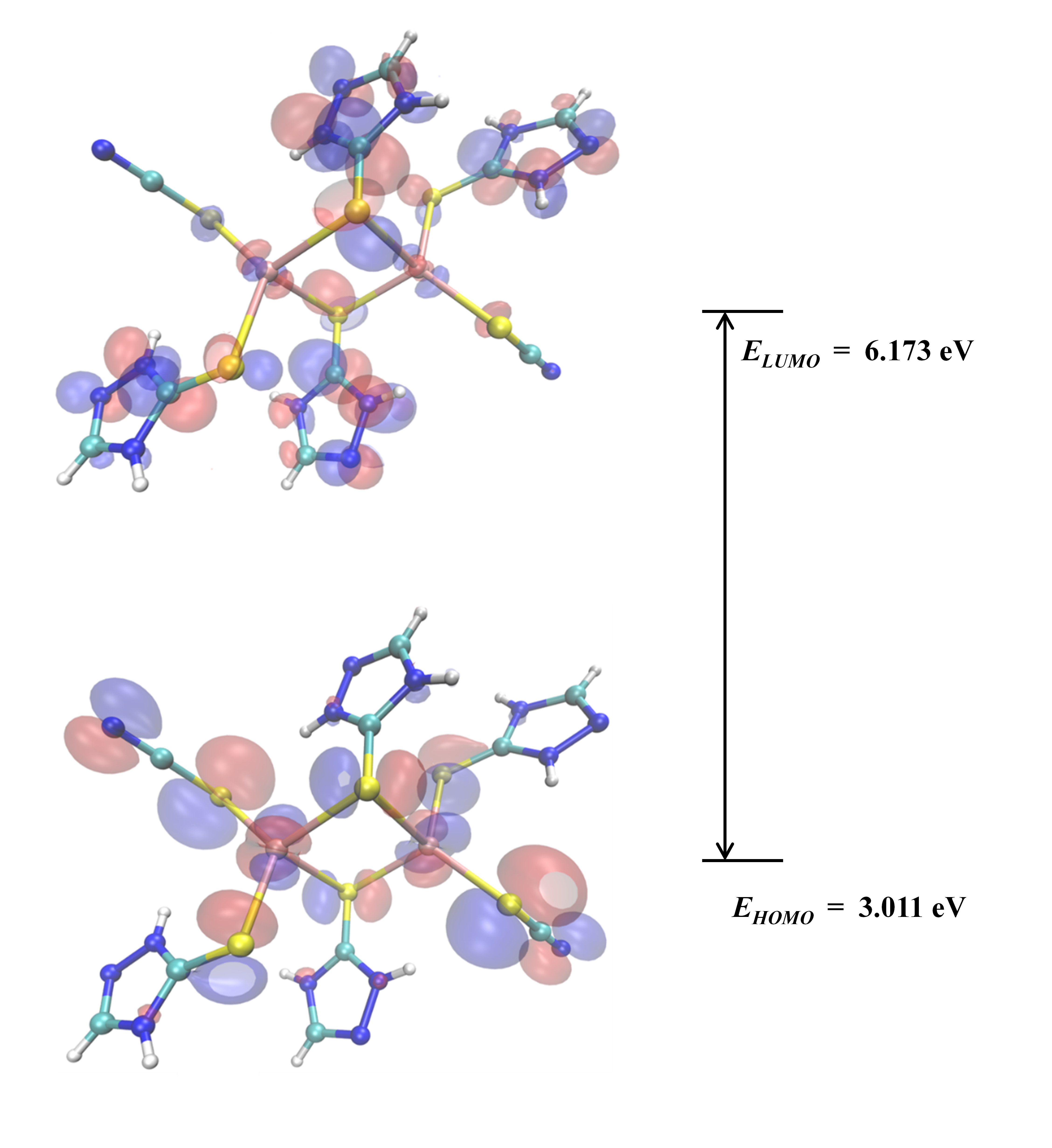

Supplement: Supplementary file 3 [file e-76-00042-sup3.tif]
